# Assessing Social Determinants of Health Among Patients With Depression at Primary Healthcare Centers in Riyadh City

**DOI:** 10.7759/cureus.48854

**Published:** 2023-11-15

**Authors:** Abdulrahman Alqahtani, Noara Alhusseini

**Affiliations:** 1 Family and Community Medicine, Alfaisal University College of Medicine, Riyadh, SAU; 2 Public Health, Ministry of Health, Riyadh, SAU; 3 Epidemiology and Public Health, Alfaisal University College of Medicine, Riyadh, SAU

**Keywords:** primary healthcare centers, saudi arabia, determinants of health, social, depression

## Abstract

Background and purpose: Depression is a common and serious medical illness that is greatly influenced by socioeconomic, demographic, and biological factors. The recognition of social determinants of depression can help reduce the risk factors and promote mental health. The purpose of this study was to assess the social determinants of health (SDOH) of patients with depression and to classify its categories at primary healthcare centers (PHCs) in Riyadh, Saudi Arabia.

Methodology: A cross-sectional study using a self-administered questionnaire was conducted among 322 participants who were selected through a convenience sampling technique from June 2021 to August 2022. The study included Saudi and non-Saudi patients at the Ministry of Health PHCs in Riyadh, Saudi Arabia, aged 12 years and older. Independent samples of t-test and one-way ANOVA test were used to assess differences in means of numerical variables. Correlations were made by the Pearson correlation coefficient. A p-value of <0.05 was set as the level of statistical significance. Logistic regression was used to determine the main variables associated with moderate to severe depression.

Results: The findings showed 51% had minimal to mild depression, 27% had moderate depression, and 22% had moderately severe to severe depression. A greater proportion of females had moderate to severe depression (61%) (p=0.007). Also, moderate to severe depression was higher in those with lower monthly income(54%)(p=0.03). Saudi nationals were found to have a higher percentage of moderate to severe depression(51%)(p=0.007). Marital status was found to be associated with depression as single respondents were more likely to have moderate to severe depression (p=0.052) with 54%.

Conclusion: SDOH including gender, income, marital status, and nationality are associated with depression. Having a comprehensive system focusing not only on mental health services but also on the social determinants of mental health is very important. Future research is needed to understand the association between depression and SDOH in Saudi Arabia. The study results can help policymakers determine the areas that require improvements.

## Introduction

Mental illness is a significant public health problem that requires attention from people in charge. Mental health disorders are a concern for people of all ages, from early childhood through old age. Many conditions fall under the area of mental illness, including depression, schizophrenia, bipolar disorder, obsessive-compulsive disorder, panic disorder, posttraumatic stress disorder, personality disorder, eating disorders, and addictive behaviors. These illnesses receive a high public health concern because they affect individuals of different ages, ethnicities, cultures, religions, and incomes. Mental health requires public health interventions such as raising awareness of the importance of mental health, along with establishing mental health screening and treatment services to reduce the burden of the disease [1].

Depression is defined as the presence of feelings of sadness, emptiness, or irritability that lasts for a long period of time and has an impact on the functioning of daily life [2]. Symptoms of depression include loss of interest and enjoyment, low energy, and lower activity for at least two weeks. People with depression have also reported anxiety symptoms, disturbed sleep and appetite, feelings of guilt and low self-worth, and difficulty concentrating [3]. Depression is influenced by factors including genetics, physiology, and psychology [3]. Social inequalities, income, education, and access to healthcare play a huge role in determining mental health status [4]. According to studies, depression is one of the treatable mental illnesses; 80-90% of people with depression get treated with a high response to the medication received [5].

According to the World Health Organization (WHO), more than 264 million people suffer from depression around the world [4]. The Centers for Disease Control and Prevention (CDC) indicated in 2016 that depression is estimated to cause 200 million lost workdays each year at a cost to employers worldwide [3]. In Saudi Arabia, there is a high prevalence of mental health disorders. The prevalence of mental health illnesses including depression and anxiety among primary healthcare center (PHC) visitors is 60% [6]. According to a study conducted on depression and social factors in Saudi Arabia that enrolled 477 people, 49.9% of them had depressive symptoms. Depression was higher among females and higher educational levels based on the results [7]. In 1983, the Ministry of Health (MOH) in Saudi Arabia began to provide psychiatric services by developing 21 hospitals with mental health services. Many patients suffering from depression may not seek help from mental health professionals. Therefore, it is very important to screen for mental health at PHCs, which could ensure covering the physical and mental needs of the patients. According to the CDC, depression screening and treatment with psychotherapy and medications are highly recommended since 80% of patients with depression show improvement after treatment [3]. In the United States, about 20% of children and adolescents suffer from mental health disorders during their lifetime. Mental health disorders among children and adolescents can lead to school failure. It is also estimated that approximately 17% of adults in the United States face mental disorders throughout their lives [8].

Work productivity is influenced by the status of mental health as people with depression tend to be less productive [3]. A study that focuses on depression in the workplace showed a 6.1% increase in productivity and 22.8% fewer absences over two years for individuals suffering from depression who received regular phone calls to encourage them to continue with depression treatment and medication. Employers saved over $2600 every year for each employee who received guidance on depression management. CDC recommends implementing a health policy that focuses on the worksite lifestyle programs such as physical activity, which could help in both preventing as well as treating depression [9].

As part of the Vision 2030 of Saudi Arabia, the MOH started to develop a new comprehensive healthcare system that covers the health of people socially, mentally, and physically through a new patient‐centered Model of Care [10]. This new model aims to ensure the coverage of all services to ensure the needs of patients are covered. The general administration of chronic diseases and health programs at the MOH spends efforts on mental health. The mental health services at the PHCs are run as part of the Vision 2030 plan, stakeholders developed a national mental health strategy that ensures the integration of healthcare services [11]. Such a strategy will help in addressing the gaps in the healthcare system as well as providing services that cover the quality, accessibility, and integration of care. The MOH in Saudi Arabia established the primary mental health program in 2016 to screen people for the most common mental health illnesses including depression and anxiety [12]. The integration of mental health services in primary healthcare settings increases the opportunity of screening and treating more people within the community. The MOH provides primary mental health services in over 1000 PHCs throughout the regions of Saudi Arabia [12]. This study focuses on the association between depression and social determinants of health in the city of Riyadh, Saudi Arabia.

## Materials and methods

This was a cross-sectional study carried out in 30 PHCs in Riyadh city, Saudi Arabia, from June 2021 to August 2021. A total of 322 Saudi and non-Saudi PHC patients aged 12 years and older were enrolled in the study. The target population of the study was PHC visitors in Riyadh, Saudi Arabia, with an estimated population of 8.4 million adults. The sample size of the study was 384. Convenience sampling was used, with confidence levels of 95% and 5% confidence intervals. Inclusion criteria were age ≥ 12 years and patients of MOH PHCs in Riyadh city. Exclusion criteria were age less than 12 years old and not a patient of MOH PHCs.

Data collection took place between June 2021 and August 2021 and was collected through an anonymous questionnaire. No personal information was revealed under any circumstances and the participation was voluntary. The questionnaire was distributed in paper form in the chosen PHCs across the city of Riyadh. The PHCs from where the data were collected were located in different regions of Riyadh city. The questionnaire was filled by healthcare providers at the PHCs by asking the patients the assigned questions to ensure complete answers to all questions.

The questionnaire consisted of two parts: the demographic questions, which included eight questions, followed by both Arabic and English versions of the Patient Health Questionnaire-9 (PHQ-9), which has nine questions related to depression. The first section included demographic questions including age, gender, income, marital status, and educational level. The second section included the PHQ-9. The PHQ-9 is used by primary care physicians and consists of a nine-item depression scale. The nine items are designed based on the diagnostic criteria for major depressive disorder. The PHQ can be used as a screening tool, and help in diagnosis along with a symptom tracking tool that can help track the severity of a patient's overall depression and can determine the improvement of specific symptoms with treatment.

The PHQ-9 tool is validated and reliable according to studies [13]. The validity of the PHQ-9 was determined in studies that were conducted in eight primary care and seven obstetrical clinics. PHQ-9 scores of >10 had a sensitivity of 88% and a specificity of 88% for major depressive disorder. The reliability and validity of the questionnaire have shown psychometric properties. It is a simple, rapid, effective, and reliable tool for screening and evaluating the severity of depression among PHC visitors. Table 1 shows the PHQ-9 scoring measures.

**Table 1:** The PHQ-9 scoring measures PHQ-9: Patient Health Questionnaire-9

Depression categories	Total score
Minimal depression	0-4
Mild depression	5-9
Moderate depression	10-14
Moderately severe	15-19
Severe	20-27

Data analysis and management

Data were entered and analyzed using Microsoft Excel 365 (Microsoft Corporation, Redmond, Washington, United States) and IBM SPSS Statistics for Windows, Version 23.0 (Released 2015; IBM Corp., Armonk, New York, United States). Both descriptive and inferential statistical analyses were applied. For categorical variables, numbers and percentages were used for expression, and for numerical variables, median, mean, and standard deviation were employed. Independent samples t-test and one-way ANOVA test were used to assess differences in means of numerical and categorical variables. Correlations were found by the Pearson correlation coefficient. A p-value of <0.05 was set as the level of statistical significance.

## Results

**Figure 1:**
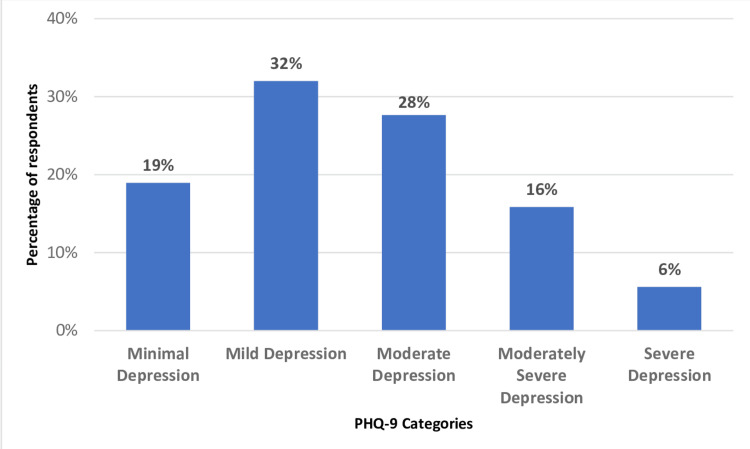
Distribution of PHQ-9 categories among the respondents (N=322) PHQ-9: Patient Health Questionnaire-9

A total of 322 respondents were included in the study. Of these, 230 (71%) were males, most of them, i.e. 173 (54%), were aged 25-34 years and another 89 (28%) were aged 18 to 24 years; 222 (69%) respondents were single (Table 2). There were 166 (52%) who had a graduate degree and another 32 (10%) had a postgraduate degree; 206 (64%) were employed while 78 (24%) were students. The monthly income of the majority, i.e. 194 (60%) was up to Saudi Riyal (SAR) 10,000 (US$ 2660) and almost all, i.e. 299 (93%), were Saudi nationals. There were 164 (51%) who had minimal to mild depression, 89 (28%) had moderate depression, and 69 (22%) had moderately severe to severe depression (Figure 1).

**Table 2:** Demographic characteristics of the respondents (N=322) SAR: Saudi Riyal

Characteristics		N	%
Age group (yrs)	18-24	89	28%
	25-34	173	54%
	35-45	41	13%
	>45	19	6%
Gender	Male	230	71%
	Female	92	29%
Marital status	Married	88	27%
	Single	222	69%
	Divorced	12	4%
Highest education level	Up to High School	124	39%
	Graduate	166	52%
	Postgraduate	32	10%
Employment status	Unemployed / Retired	38	12%
	Student	78	24%
	Employed	206	64%
Monthly Income	Up to 10,000 SAR	194	60%
	>10,000 to <15,000 SAR	81	25%
	15,000+ SAR	47	15%
Nationality	Saudi	299	93%
	Non-Saudi	23	7%

The depression categories according to the PHQ-9 were categorized into two groups: Minimal/Mild depression (n=164) and Moderate to Severe depression (n=158). These were compared by the demographic variables as shown in Table 3. It was seen that a greater proportion of females (61%) had moderate to severe depression as compared to 44% of males (p=0.007). Also, moderate to severe depression was higher in those with a monthly income of less than 10,000 SAR (54%) as compared to 32% in those with income >SAR 15,000 (p=0.03). Saudi nationals were found to have a higher (51%) percentage of moderate to severe depression as compared to non-Saudis in whom it was 22% (p=0.007). Marital status was found to have a borderline significant association (p=0.052) with 54% of the single respondents having moderate to severe depression as compared to 39% of the married respondents. There was no significant association found between the depression category and age group (p=0.31), education level (p=0.81), employment status (p=0.23), and residence status (p=0.13).

**Table 3:** Association of demographic variables with depression categories PHQ-9: Patient Health Questionnaire-9; SAR: Saudi Riyal

			PHQ-9 (two categories)				
			Minimal/Mild Depression		Moderate to Severe Depression		
		n	n	%	n	%	p-value
Age group	18-24 years	89	52	58%	37	42%	0.31
	25-35 years	73	82	47%	91	53%	
	35-45 years	41	19	46%	22	54%	
	>45 years	19	11	58%	8	42%	
Gender	Male	230	128	56%	102	44%	0.007*
	Female	92	36	39%	56	61%	
Marital status	Married	88	54	61%	34	39%	0.052
	Single	222	103	46%	119	54%	
	Divorced	12	7	58%	5	42%	
Highest education level	Up to High School	124	62	50%	62	50%	0.81
	Graduate	166	84	51%	82	49%	
	Postgraduate	32	18	56%	14	44%	
Residence status	Own place	209	113	54%	96	46%	0.13
	Renting	113	51	45%	62	55%	
Employment status	Unemployed / Retired	38	15	40%	23	60%	0.23
	Student	78	44	56%	34	44%	
	Employed	206	105	51%	101	49%	
Monthly Income (SAR)	Up to 10,000	194	90	46%	104	54%	0.03*
	>10,000 to <15,000	81	42	52%	39	48%	
	>15,000	47	32	68%	15	32%	
Nationality	Saudi	299	146	49%	153	51%	0.007*
	Non-Saudi	23	18	78%	5	22%	

Table 4 shows the results of the logistic regression to determine the main variables associated with moderate to severe depression. All the variables found to be significantly associated with moderate to severe depression (gender, income, nationality) as well as marital status and residence status were included in the logistic regression. The results showed that being female had an adjusted odds ratio (aOR) of 2.30 (95%CI 1.32-4.00; p=0.003) as compared to males. Respondents who were single were found to have an aOR of 1.80 (95%CI 1.004-3.21; p=0.048) as compared to married respondents. Those with income less than 10,000 SAR had an aOR of 2.18 (95%CI 1.06-4.46; p=0.03) as compared to those with income greater than SAR 15,000. Saudi nationals were found to have an aOR of 3.28 (95%CI 1.10-9.75; p=0.03) as compared to non-Saudis.

**Table 4:** Logistic regression for variables associated with moderate to severe depression AOR: adjusted odds ratio; SAR: Saudi Riyal *significant at p<0.05

				95% CI for AOR		
		n	AOR	Lower	Upper	p-value
Gender	Male	230	1			
	Female	92	2.30	1.32	4.00	0.003*
Marital Status	Married	88	1			
	Single	222	1.80	1.004	3.21	0.048*
	Divorced	12	1.05	0.26	4.15	0.95
Residence	Own place	209	1			
	Rented	113	1.56	0.95	2.58	0.08
Income (SAR)	15,000+	47	1			
	>10,000 to <15,000	81	1.56	0.70	3.49	0.28
	Up to 10,000	194	2.18	1.06	4.46	0.034*
Nationality	Non-Saudi	23	1			
	Saudi	299	3.28	1.10	9.75	0.032*

## Discussion

To the best of our knowledge, this is the first study in Riyadh, Saudi Arabia, to examine the relationship between depression and social determinants of health (SDOH) and lifestyle factors in PHCs. Depression is one of the most common mental health conditions worldwide and has been one of the main causes of disabilities [4]. Public health interventions that address depression in Saudi Arabia have been established by the MOH including raising awareness of depression and mental health in the general population and early detection of depression among PHC visitors. The study concluded that there was an association between depression and gender, income, living conditions, and marital status. It is possible that changes that occur in people’s lives, e.g. retirement, increased isolation, the death of loved ones, financial struggle, and other cultural differences, could contribute to increased depression prevalence. According to AlKhathami et al.'s study in the Eastern region of Saudi Arabia, the prevalence of mental health problems including depression and anxiety in PHCs is 60% [6]. Depression is a multifactorial condition that is impacted by biological and environmental factors. The current study focuses on a number of factors including gender, age, employment status, income, marital status, educational status, and nationality.

The present study showed that females are more likely to have depression compared to males. A systematic review conducted in Canada that focused on gender and mental health agreed with our conclusion that women are more likely to suffer from depression compared to their male counterparts [14]. Females go through hormonal changes, along with the stress of pregnancy for some women, which can increase the likelihood of developing mental health disorders such as depression. In contrast, a cross-sectional study conducted in Saudi Arabia showed that men were more likely to develop depression compared to females (p-value <0.001) [15]. This could be because there were fewer men who participated in the study as the female-to-male ratio was 2:1.

According to the results of the current study, there was no significant association between the depression category and the age groups (p=0.31). Another study that focused on mental health during the coronavirus disease 2019 (COVID-19) pandemic and lockdown concluded that younger adults (<35 years) were more likely (p-value <.05) to suffer from mental health issues compared to other age groups. This could be linked to the fact that younger adults are used to a certain active routine and the lockdown may have meant more restrictions on their lifestyle leading to developing mental health disorders [16]. Another important finding of a Portuguese study is that the age group of 40-64 years was more likely to suffer from depression compared to other age groups [17]. This could be because this age group has more responsibilities than other age groups such as being in charge of taking care of their kids and work [17,18].

According to the present study, there was no significant association between the depression category and employment status (p=0.23). That might be due to the fact that there was a diversity in the age group of the study participants as there were students, workers, and unemployed enrolled in the study. In support of this, a study conducted in Mexico concluded that employment status is not associated with the presence of depressive symptoms in women [19]. In contrast, a study conducted in Canada that focused on social determinants of mental health showed that being unemployed impacts the health of individuals, including mental health. The link between unemployment and depression could be because of the financial struggle unemployed individuals go through, which can impact someone’s mental health [20]. A systemic review focusing on depression and employment status worldwide concluded that unemployment can lead to a higher prevalence of depressive symptoms and major depressive disorder [21]. Supporting this result, a study was conducted to measure the association between depression and employment status among caregivers, and the results concluded that nonworking caregivers were more likely to suffer from depression compared to working caregivers [22].

Saudi nationals were found to have a higher percentage (51%) of moderate to severe depression as compared to non-Saudis in whom it was 22% (p=0.007). This could be because most of the participants in our study were Saudis. According to the current study, severe depression was higher in those with a monthly income of less than 10,000 SAR (54%) as compared to those with income >SAR 15,000 (32%) (p=0.03). We believe this could be because the likelihood of developing depression may be higher in individuals facing financial obstacles compared to those who are financially stable.

Financial struggle is very common among university students. A cross-sectional study in Jordan that included 1582 undergraduate students concluded that students’ income tends to be a risk factor for anxiety and depression [23]. Similar results were shown by another study among public university students that suggests that there is psychological distress among students from lower socioeconomic groups living in the United States during the COVID-19 pandemic [24]. A study conducted in Thailand to address the financial burden during COVID-19 supports this result as the study determined that there was an association between economic burden, especially self-reported financial problems, and adverse mental health outcomes [25]. According to a systematic review that included 64 studies, low and middle-income communities have been linked to depression. The study focused on antenatal depression [26].

There was no significant association between depression and education level (p=0.81). A study focusing on factors affecting depression during pregnancy showed different results. The study concluded that factors such as the educational level of pregnant women play an important role in the severity of depression [27]. Education makes people more aware of the things that trigger their mental health condition; therefore, they can avoid triggers and have better control over their mental health journey compared to those who are less educated about their health [28]. A study focusing on depression among hospitalized patients with chronic conditions showed that mental health distress was higher among educated patients; this could be because educated patients are more familiar with their health condition and more knowledgeable about the complications of their condition [28].

Marital status was found to be one of the risk factors for depression, especially among those who are single [28]. Our study results showed a borderline significant association (p=0.052) with 54% of single participants suffering from depression as compared to 39% of married participants. This could be due to the loneliness felt by single individuals along with the lack of emotional support that some married people have. Moreover, a study conducted in Taiwan revealed that being divorced can put people at a higher risk of suffering from depression. Divorce is a sensitive matter for many people and it can be very frustrating, especially for those who rely emotionally on their partners [29]. A study focusing on marital status and depression concluded that separated/divorced/widowed/never-married middle-aged and elderly individuals might be at a high risk of facing depression during their lifetime [30]. In conclusion, individuals with unstable marital statuses are more likely to be depressed. In particular, unstable marital status could lead to financial decline which could result in raising the chance of developing depression [29].

Our study results concluded that there was no significant association between the depression category and residence status (p=0.13) This can be supported by the results of another study conducted on depression and sociodemographic factors. The study results showed that depression is not linked to the living conditions of individuals. A study that included college students living in poor conditions concluded that depression and anxiety are more common among college students living in a poor areas compared to those who live in better areas [31]. Another research conducted on the association between depressive mood and living conditions showed that poor housing quality, noise, and air pollution could put individuals living in such an environment at higher risk of facing depressive moods [32]. Living conditions and environmental pollution are risk factors for depression and must be taken into account during planning to prevent depression [32].

Recommendations

According to the findings of the results, SDOH play an important role in affecting depression. The status of depression affects the wellness of the entire body; therefore, the government of Saudi Arabia established the Primary Mental Health Program which screens, treats, and refers patients. Our study has revealed that gender, income, and marital status are related to depression. This is a call for the stakeholders to interfere and start paying attention to the social determinants of depression listed above. Stakeholders such as healthcare providers, public health specialists, and policymakers are highly encouraged to focus on raising awareness of depression and how our lifestyle including our age, income, educational level, and gender affect our mental health. Social media is a very important tool used in our daily routine and has a great impact on our behavior; therefore, establishing a depression awareness campaign through social media channels can be a very effective step. Depression has a huge stigma in our community and that can worsen the situation of depression for many individuals. Social media awareness campaigns on depression can decrease the spread of the stigma linked to depression. Early interventions such as school campaigns focused on raising awareness of depression will help in reducing the stigma of depression. Since there has already been a system that screens people for depression at PHCs, the MOH can apply a checklist to determine which patients applied to the list of social determinants of depression. If we can determine the income, marital status, and gender of patients, we can ensure that those patients receive special care and get referred to the proper healthcare service.

Strengths and limitations

The study was conducted among MOH PHC patients. This is the first study to our knowledge that focuses on depression and SDOH among PHC patients in Riyadh City. This study is novel as it will add new information to the current literature about depression and SDOH.

Since this research was conducted among PHC patients only, we were required to distribute the questionnaire among the PHCs that provided mental health services. A limited period of time was given for data collection. This, along with the fact that not all PHCs provide mental health services, made our journey more challenging. The cross-sectional nature of the study limits any causal inferences. The PHQ-9 is not considered a diagnostic tool for depression. Since the sample was conducted among PHCs under the MOH, all private clinics were excluded; hence, the results cannot be generalized to the general population. This study was conducted during the COVID-19 pandemic. It could have had an impact on the study population causing unforeseen bias since COVID-19 can affect someone’s mental health status. Another factor to consider is the gender since there was a significant difference with only 30% females and 70% males, which could impact the result of the study.

## Conclusions

This study aimed to assess social determinants of health among depression patients at PHCs in Riyadh, Saudi Arabia. Depression has been a public health concern worldwide as the prevalence has increased according to the WHO. Our results have shown that females, people with a monthly income of less than 10,000 SAR, divorced individuals, and Saudi people were associated with moderate to severe depression, whereas age, living condition, and educational level were not linked to depression. The MOH in Saudi Arabia pays special attention to the complexity of mental health among the population. Depression is screened and treated in both primary and secondary care. Despite the great efforts to limit the severity of mental health in Saudi Arabia, focusing on the SDOH related to depression is essential to have a comprehensive system that can fill the gaps present today in the mental health care system.
